# A hierarchical opportunistic screening model for osteoporosis using machine learning applied to clinical data and CT images

**DOI:** 10.1186/s12859-022-04596-z

**Published:** 2022-02-10

**Authors:** Liyu Liu, Meng Si, Hecheng Ma, Menglin Cong, Quanzheng Xu, Qinghua Sun, Weiming Wu, Cong Wang, Michael J. Fagan, Luis A. J. Mur, Qing Yang, Bing Ji

**Affiliations:** 1 School of Control Science and Engineering, Shandong University, 17923 Jingshi Road, Jinan, Shandong People’s Republic of China; 2 Department of Orthopedics, Qilu Hospital of Shandong University, Jinan, Shandong People’s Republic of China; 3 School of Engineering, University of Hull, Hull, HU6 7RX UK; 4 Institute of Biological, Environmental and Rural Sciences (IBERS), Aberystwyth University, Aberystwyth, Wales UK; 5 Department of Breast and Thyroid, Shandong Provincial Hospital Affiliated to Shandong First Medical University, Jinan, Shandong People’s Republic of China

**Keywords:** Osteoporosis, Opportunistic screening, Machine learning, Clinical data, CT

## Abstract

**Background:**

Osteoporosis is a common metabolic skeletal disease and usually lacks obvious symptoms. Many individuals are not diagnosed until osteoporotic fractures occur. Bone mineral density (BMD) measured by dual-energy X-ray absorptiometry (DXA) is the gold standard for osteoporosis detection. However, only a limited percentage of people with osteoporosis risks undergo the DXA test. As a result, it is vital to develop methods to identify individuals at-risk based on methods other than DXA.

**Results:**

We proposed a hierarchical model with three layers to detect osteoporosis using clinical data (including demographic characteristics and routine laboratory tests data) and CT images covering lumbar vertebral bodies rather than DXA data via machine learning. 2210 individuals over age 40 were collected retrospectively, among which 246 individuals’ clinical data and CT images are both available. Irrelevant and redundant features were removed via statistical analysis. Consequently, 28 features, including 16 clinical data and 12 texture features demonstrated statistically significant differences (*p* < 0.05) between osteoporosis and normal groups. Six machine learning algorithms including logistic regression (LR), support vector machine with radial-basis function kernel, artificial neural network, random forests, eXtreme Gradient Boosting and Stacking that combined the above five classifiers were employed as classifiers to assess the performances of the model. Furthermore, to diminish the influence of data partitioning, the dataset was randomly split into training and test set with stratified sampling repeated five times. The results demonstrated that the hierarchical model based on LR showed better performances with an area under the receiver operating characteristic curve of 0.818, 0.838, and 0.962 for three layers, respectively in distinguishing individuals with osteoporosis and normal BMD.

**Conclusions:**

The proposed model showed great potential in opportunistic screening for osteoporosis without additional expense. It is hoped that this model could serve to detect osteoporosis as early as possible and thereby prevent serious complications of osteoporosis, such as osteoporosis fractures.

**Supplementary Information:**

The online version contains supplementary material available at 10.1186/s12859-022-04596-z.

## Introduction

Osteoporosis is a common metabolic skeletal disease, occurring primarily in post-menopausal women and older men [1]. Osteoporosis leads to decreased bone mineral density (BMD) and changed bone microarchitecture, consequently increasing bone fragility and fracture risk [2]. With an aging population, the number of hip fractures caused by osteoporosis is predicted to reach about 6 million worldwide by 2050 [3].

DXA is regarded as the “gold standard” for osteoporosis detection in clinics [4]. Prospective studies indicated that each standard deviation decrease in BMD can lead to a 1.5 to 2.5-fold risk of fracture [5]. As a result, it is recommended that women aged over 65, man over 70, and younger individuals with risk factors of osteoporosis should be screened for osteoporosis and take a DXA test [6]. However, only a limited percentage of these people are screened using DXA due to its relatively high cost, the risk of ionizing-radiation, and insufficient awareness [7–9]. As a result, the detection and treatment rates of osteoporosis remain low. Osteoporosis usually lacks obvious symptoms and many individuals are not diagnosed until osteoporotic fractures occur [10]. Therefore, it is vital to develop methods to identify individuals at-risk based on methods other than DXA.

In addition to BMD measurement, clinical risk factors are important in osteoporosis assessment [11]. Indeed, a series of tools have been developed to predict osteoporosis risk based on clinical risk factors [12]. The international osteoporosis foundation proposed a one-minute osteoporosis risk awareness test utilizing such as low body mass index (BMI), vitamin D deficiency, and poor nutrition to access potential osteoporosis risks [13]. Similarly, osteoporosis self-assessment tool (OST), osteoporosis risk assessment instrument (ORAI), simple calculated osteoporosis risk estimation (SCORE), and osteoporosis index of risk (OSIRIS) were developed to predict osteoporosis risk [14–17]. Other clinical predictive tools have also been proposed to predict osteoporotic fracture risk, such as FRAX, Garvan, and QFracture [18]. These methods usually utilize 2 to 30 clinical risk factors, among which age, weight, and history of fracture were used most frequently [18]. Recently, some researchers have attempted to use machine learning (ML) methods to increase the accuracy of osteoporosis risk prediction based on clinical risk factors. These models go beyond the linear or nonlinear combination of all the input risk factors and have the potential to capture underlying trends and patterns, which is impossible for the tools mentioned above [19]. Yoo, et al. [20] compared the performance of several ML methods, including supporting vector machine (SVM), random forests (RF), artificial neural network (ANN), and logistic regression (LR), with traditional tools (OST, ORAI, SCORE, and OSIRIS) in identifying postmenopausal women at risk of osteoporosis. The results showed that SVM was more effective than traditional tools and other ML methods mentioned above. de Lira, et al. [21] used J48 decision tree algorithm to discriminate between osteoporosis and osteopenia in women via BMI, age, menopause status, and other risk factors and obtained an AUC of 0.65. Besides these demographic characteristics, other studies [22, 23] also considered several routine laboratory tests data, including alkaline phosphatase, calcium, phosphorus, the numbers of hemoglobin and lymphocyte and albumin to predict osteoporosis risk. Such approaches underline the benefits of routine laboratory tests data in identifying osteoporosis risk.

Medical imaging also shows great potential in osteoporosis prediction. Kawashima, et al. [24] found a potential utility in the differences of texture features derived from non-contrast head CTs between individuals with and without osteoporosis. Mookiah, et al. [25] differentiated healthy and osteoporotic fracture individuals based on texture features extracted from CT images with a classification accuracy of 83%. This work showed the feasibility of opportunistic osteoporosis screening by texture analysis of CT images. Valentinitsch, et al. [26] utilized 3D texture features and regional volumetric BMD obtained from CT images for opportunistic osteoporosis screening. RF classifier was used and achieved an AUC of 0.88 in identifying individuals with and without osteoporosis. In addition to CT images, X-ray and MRI images have also been utilized to assess the risk of osteoporosis and osteoporotic fractures [7, 27, 28].

Thus, the individual potential of assessing osteoporosis risk factors, medical images, and routine laboratory tests data to predict osteoporosis risk is well-established. However, to our knowledge, systematic studies have not accessed osteoporotic risk by combining osteoporosis risk factors, routine laboratory tests data, and medical images together. In this context, we attempt to construct a hierarchical model to identify individuals with osteoporosis as an alternative approach to a DXA test. The model consisted of three layers based on the popularity of test people usually underwent. To be specific, the first layer utilized demographic characteristics only, with clinical data (including demographic characteristics and routine laboratory tests data) for the second layer, and clinical data together with CT images that partly or completely cover the spine for the third layer. Six machine learning algorithms, including LR, SVM, ANN, RF, eXtreme Gradient Boosting (XGBoost) and Stacking that combined the aforementioned other five models, were successively used as classifiers to discriminate individuals between osteoporotic and non-osteoporotic.

## Materials and methods

**Fig. 1:**
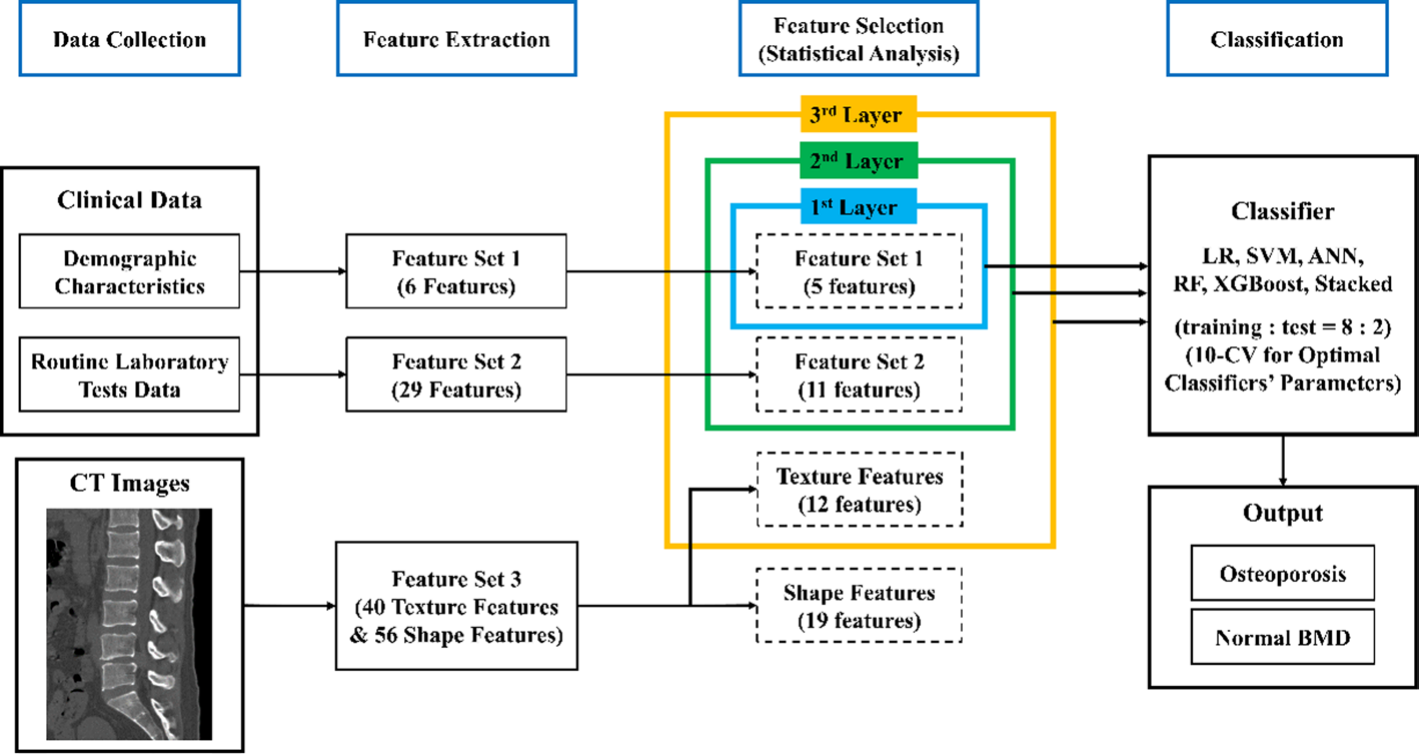
Schematic elaboration of the study design

As shown in Fig. 1, the proposed model is comprised of four main parts: data collection, features extraction, features selection and classification. In data collection, participants’ demographics characteristics, routine laboratory tests data and CT images covering lumbar spine were collected and then features were extracted from them. Statistical analysis, including Mann–Whitney U test, Chi-square test, Pearson correlation test and Kruskal–Wallis H-test, were used successively to eliminate redundant and irrelevant features. Comparing to texture features, the model based on the combination of texture and shape features didn’t achieve a better performance. Thus, the remaining features were used except for shape features as the input of the proposed model. The proposed model included three layers according to the features available. The features of three layers overlapped with each other. The higher the layer lay, more features were used. What’s more, three layers of the model worked independently, which means only one layer worked for a specific model input. The details of features for each layer were listed in Additional file 1: Table S7. The model was built based on six classifiers respectively, including LR, SVM, ANN, RF, XGBoost and Stacking. The model performances were eventually evaluated on a hold-out test set, which accounted for 20 percent of the dataset.

### Patient characteristics

**Fig. 2:**
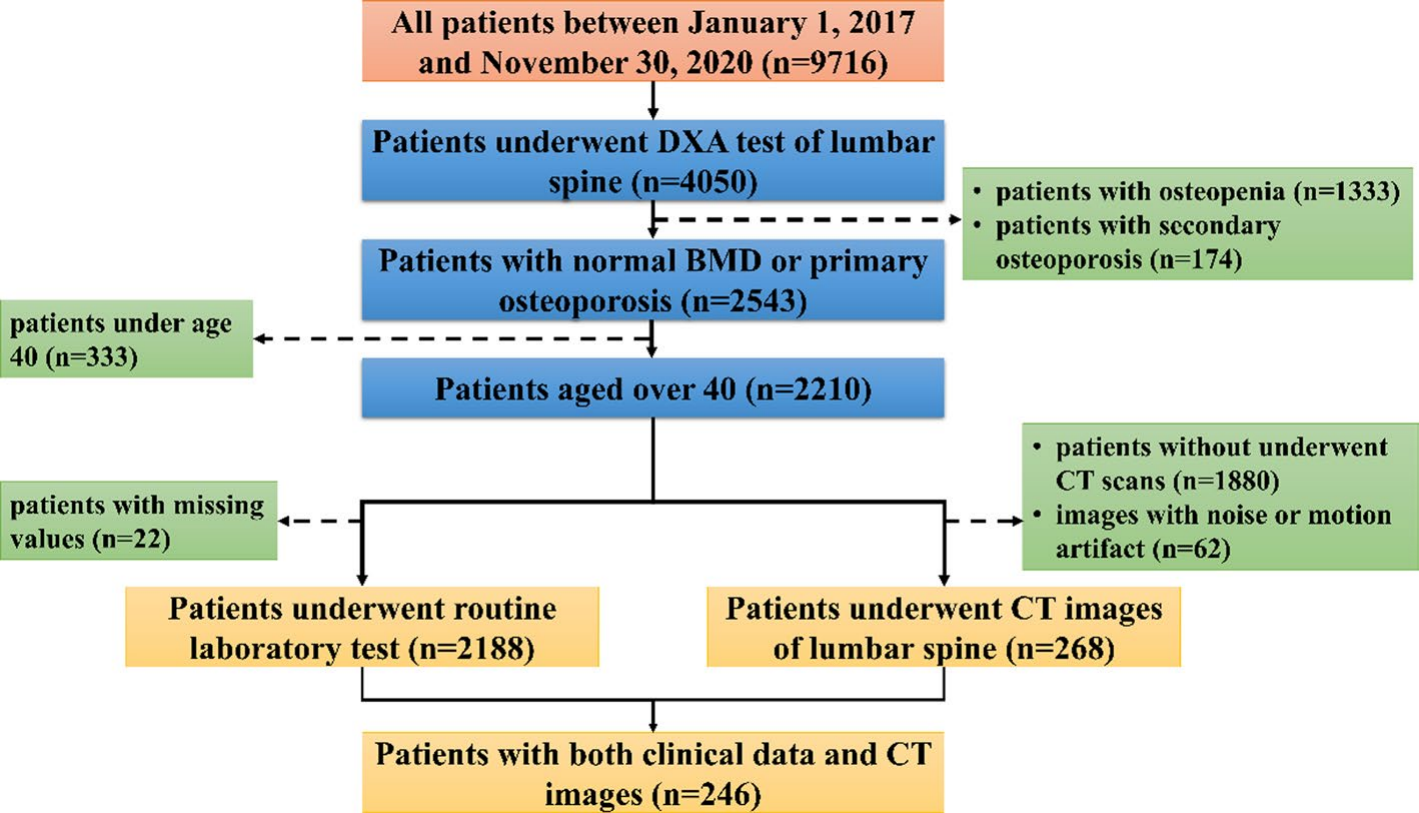
Flow chart of participants selection

We conducted a retrospective study, which complied with the World Medical Association Declaration of Helsinki. The study obtained local ethics committee approval (KYLL-2020(KS)-743) and informed consent was not required owing to the retrospective nature of the study. The individuals over age 40 who underwent DXA screening were collected from Qilu Hospital of Shandong University and Shandong Provincial Hospital. Participants’ clinical data were derived from both centers, while CT images were derived from Qilu Hospital of Shandong University only. Routine laboratory tests, CT scans, and DXA screening for an individual were performed in the same time. These individuals were classified into normal and osteoporotic groups according to T-score (for postmenopausal women and men over age 50) or Z-score (for others) based on BMD analysis report of DXA scanned lumbar spine. It is noted that patients diagnosed with secondary osteoporosis were not included in the cohort. Cases of osteopenia and cases with missing values were omitted. The flowchart of participant selection is shown in Fig. 2.

### CT data and processing

CT images covering the lumbar spine were retrieved from the PACS and saved in DICOM format. Any data with noise or motion artifacts were excluded. The images were acquired with different CT scanners (SIEMENS SOMATOM Definition AS, and SIEMENS SOMATOM Definition) with the same single collimation width of 0.6 mm. The tube voltage of images ranged from 100 to 140 kV and the slice thickness of CT scans was 1.00 mm. No contrast agents were used in CT scans. Sagittal images were reconstructed in the RadiAnt DICOM Viewer and exported as BMP format. The single mid-sagittal image of CT scans for each individual was utilized. The bone window was set to default parameters of RadiAnt (window width: 1500 and window level: 300). As the DXA test measures BMD of lumbar vertebral bodies L1-L4, the same regions of median sagittal images were segmented manually as ROIs. It’s noted that fractured vertebral bodies were discarded and only intact vertebrae were considered as ROIs. Segmented images were all resized to $64\times 64$ pixels to eliminate the influence of size, and then saved as 8-bit grayscale images.

### Feature extraction

Analysis features were extracted from the clinical data and CT images, where clinical data consisted of demographic characteristics (including age, gender, BMI, systolic pressure, diastolic pressure, pulse pressure, and menopause status) and routine laboratory tests data (comprising complete blood count, renal and liver function test, blood sugar test and lipid blood test). In total, 35 features were extracted from the clinical data; their details are included in Additional file 1: Table S1. Furthermore, gender information of individuals is included in the indicator of MPS rather than as a separate indicator to avoid redundancy. To be specific, MPS has three statuses, corresponding to women in menopause, women not in menopause or men, respectively.

Texture data of CT images has previously been shown to be useful in osteoporosis identification [24–26], as has shape information [29]. Hence texture and shape features were extracted from ROIs as descriptors of the CT images. Texture features were extracted from each ROI of cortical and cancellous bone to fit DXA [30]. Texture features consisted of five Gray-Level Co-occurrence Matrix (GLCM) parameters, 4 Gray-Level Gradient Matrix (GLGM) parameters, and 6 Gray-Level Histogram (HI) parameters. GLCM proposed by Haralick, et al. [31] included 14 parameters in total, of which the 5 most widely-used parameters were chosen for this study, namely entropy, energy, contrast, correlation, and homogeneity [24]. The given GLCM distance was set to one in all four directions, and the number of gray levels was 256. In order to minimize directional ambiguity, the mean and standard deviation of GLCM parameters in four orientations (0°, 45°, 90°, and 135°) were computed [26]. Additionally, gradient information was employed in GLGM that included mean, variance, skewness, and kurtosis [24]. HI incorporated the gray-level information of the image and mean, variance, skewness, kurtosis, energy, and entropy were used as its parameters.

**Fig. 3:**
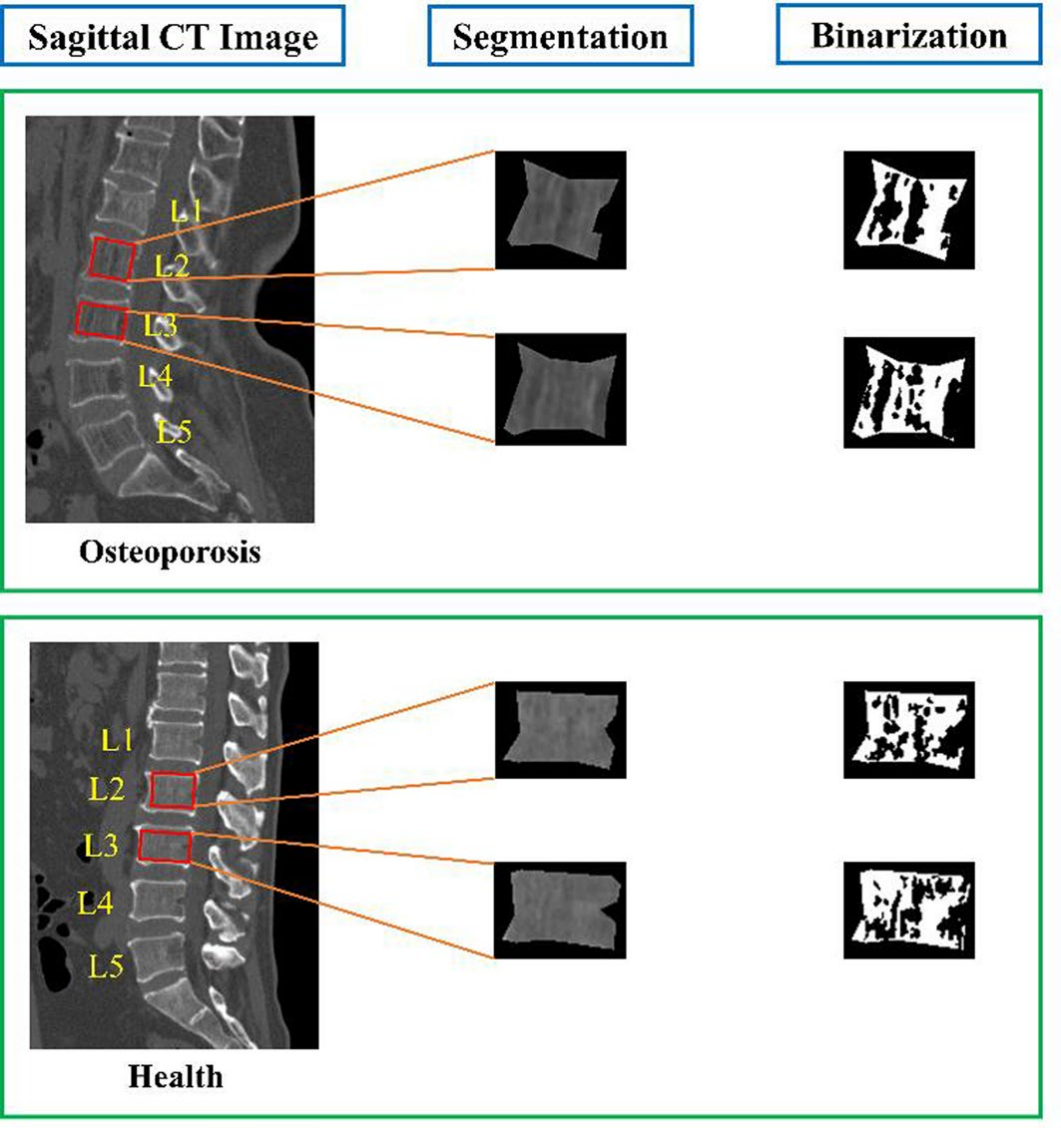
Sagittal CT images of participants with osteoporosis and normal BMD

Trabecular microarchitecture has been proven to be a determinate of bone strength [32] and the inter-trabecular space expands as osteoporosis progresses [23]. Figure 3 demonstrated the differences between individuals with and without osteoporosis in CT images. It’s obvious that the individual with osteoporosis has larger inter-trabecular space and more notable permeability of vertebral body than the health. Shape parameters describing the outline of inter-trabecular space were utilized, which includes perimeter, area, regional density (the ratio of the area to the squared perimeter), circularity, solidity, length–width ratio (the aspect ratio of the regional minimum bounding rectangle), rectangularity (the ratio of the area to the area of regional minimum bounding rectangle) and 7 Hu’s invariant moments [29]. The maximum between-class variance (Otsu) method [33] was utilized to acquire binary images that represented trabecular and inter-trabecular space ahead of calculating shape features [34]. The Otsu method is subject to pixel (gray-scale) values, and cortical bone has higher pixel values than cancellous bone, which may influence the segmentation of trabecular bone, hence cortical bone was excluded from the shape analysis. furthermore, since the inter-trabecular space was usually comprised of several regions, the mean and standard deviation of shape parameters were computed for each vertebra. Additionally, lumbar vertebral bodies L1-L4 were segmented for every individual. The mean and standard deviation of each image parameter were then computed to describe the overall condition of an individual. In total, 96 image features were extracted from the CT images; their details are presented in Additional file 1: Tables S2 and S3 and the distribution of them between two groups are shown in Additional file 1: Table S4. Furthermore, only reproducible features with intraclass correlation coefficient greater than 0.8 were used [35]. The process of image features extraction mentioned above was carried out in MATLAB R2019a.

### Statistical analysis

Statistical analysis was initially used to remove redundant or irrelevant features. Firstly, Mann–Whitney U tests and Chi-square tests were utilized to assess differences between individuals with and without osteoporosis for numerical and nominal features respectively. Only features that significantly differ between the two groups were picked up. Then Pearson correlation tests were utilized to indicate the linear dependence between features. Redundant features (Pearson correlation coefficient $\left|\gamma \right|\ge 0.8$) were removed to ensure no high correlation exists in the final selected features for the model. At last, Kruskal–Wallis H-test was used to evaluate the effect of CT protocol settings on textural and shape features. *P* = 0.05 was regarded as the statistical level throughout our study. Statistical analysis was performed using IBM SPSS Statistics 24.

### Classification

LR, SVM with radial-basis function kernel, ANN, RF, XGBoost and Stacking were used as the classifier to identify individuals with osteoporosis. Specially, the first layer of the Stacking used the above five models and the second layer used LR, which only trained on the predictions of the first layer. A grid-search coupled with stratified tenfold cross validation was employed to estimate the hyper-parameters of classifiers, which was performed on training set. Moreover, as these classifiers can’t handle nominal features, One-Hot coding was utilized before model training. Min–max normalization that transforms features by scaling each feature to (0,1) was also performed for LR which is sensitive to the dimension. ROC (receiver operating characteristic) curve analysis was adopted to assess the classification performance of classifiers; ROC weights sensitivity and specificity equally and has better discriminative ability than accuracy [36, 37].

The dataset was randomly split into training and test set with a ratio of 8:2. In order to diminish the influence of data partitioning, this process was repeated five times, and the training and test set had the same class distribution as the original dataset. The model performance was then evaluated by averaging over all five randomly shuffled test sets. Feature importance was computed using coefficient of features in the decision function for LR, the average gain across all splits features was used for XGBoost and Gini importance for RF. Owing to the fact that there was not suitable property for SVM with radial-basis function kernel, ANN and Stacking to represent the importance of features, feature importance was not computed for them. The process mentioned above was performed in Pycharm-Professional-2019.2.4. The detailed packages used are listed in Additional file 1: Table S5.

## Results

### Patient characteristics and CT data

The patients’ characteristics are shown in Table 1. 2210 individuals were collected retrospectively, all of which had demographic characteristics. Among them, 2188 and 268 individuals underwent routine laboratory tests and CT scans, respectively, while 246 individuals underwent both of them. 43.1%, 47.0%, and 47.6% of patients were diagnosed as osteoporosis among each group. Statistically significant differences were found in the ages between individuals with and without osteoporosis in all groups.

**Table 1:** Demographic characteristics of each subgroup

Group	Condition	Number		Age	*P*-value
Clinical data group	Osteoporosis	943	2188	65.67 $\pm$ 9.691	0.000
	Normal	1245		55.77 $\pm$ 10.066	
Image group	Osteoporosis	126	268	65.66 $\pm$ 8.394	0.000
	Normal	142		58.61 $\pm$ 9.912	
Combination group	Osteoporosis	117	246	65.68 $\pm$ 7.976	0.000
	Normal	129		58.50 $\pm$ 10.152	

Age was expressed as mean $\pm$ standard deviation and *P*-value was used to compare the difference between individuals with osteoporosis and normal BMD in age

In the image group, the tube voltage of most cases (244/268) was 120 kV as well as others were 80 kV (21/268) and 140 kV (3/268) respectively. No significant differences were found in the tube voltage between osteoporosis and normal BMD (*p* = 0.547). However, 12 image features showed significant differences in the tube voltage and then were excluded.

### Features extraction and statistical analysis

In total, 131 features were extracted in the study, involving 35 clinical data features and 96 image features. 47 features including 16 clinical data features, 12 texture features, and 19 shape features exhibited a statistically significant difference (*p* < 0.05) between individuals with and without osteoporosis, no highly linear correlation between them, and no significant differences among tube voltage or good reproducibility. The details of selected features are listed in Additional file 1: Table S6. Additional file 1: Figure S1 showed the violin plots for each feature between two groups and Additional file 1: Figure S2 showed the correlation between features in heatmap (Pearson correlation coefficient $\upgamma$ ranged from −0.764 to 0.779).

### Classification

Table 2 shows the performances of clinical data and CT images in identifying osteoporotic individuals. The performance of demographic characteristics outperformed routine laboratory tests, and texture features outperformed shape features. Moreover, when demographic characteristics and routine laboratory tests data were combined, all these classifiers showed better classification performances. Compared with using texture features alone, the performance was not improved when texture features and shape features were both utilized. Consequently, shape features were not taken into account for the third layer of the model. Furthermore, the model based on all the classifiers showed acceptable performances and specially, Stacking showed better performances than using single model in almost each feature group.

**Table 2:** Classification performance of each classifier on clinical data and CT images

Features		LR	SVM	ANN	RF	XGBoost	Stacking	N
DC	Training	0.798	0.800	0.795	0.836	0.828	0.819	2188
	Test	0.805	0.806	0.798	0.809	0.808	**0.810**	
RLT	Training	0.696	0.725	0.702	0.856	0.828	0.819	
	Test	0.677	**0.694**	0.680	0.687	0.687	**0.694**	
CD	Training	0.815	0.837	0.818	0.898	0.893	0.872	
	Test	0.813	0.824	0.815	0.820	0.820	**0.828**	
TFs	Training	0.970	0.971	0.942	0.989	0.976	0.976	268
	Test	0.949	0.951	0.929	0.947	0.933	**0.953**	
SFs	Training	0.869	0.882	0.829	0.945	0.905	0.892	
	Test	0.855	0.875	0.850	0.867	0.853	**0.876**	
IFs*	Training	0.977	0.979	0.932	0.978	0.973	0.982	
	Test	0.950	0.957	0.931	**0.960**	0.938	0.959	

*DC* Demographic Characteristics; *RLT* Routine Laboratory Tests; *CD* Clinical Data; *TFs* Texture Features; *SFs* Shape Features; *IFs* Image Features

The performances of each classifier were evaluated by the mean of five repeated experiments

The highest values among the six classifiers for each feature set in test set were highlighted in bold

*Image features included texture and shape features

**Fig. 4:**
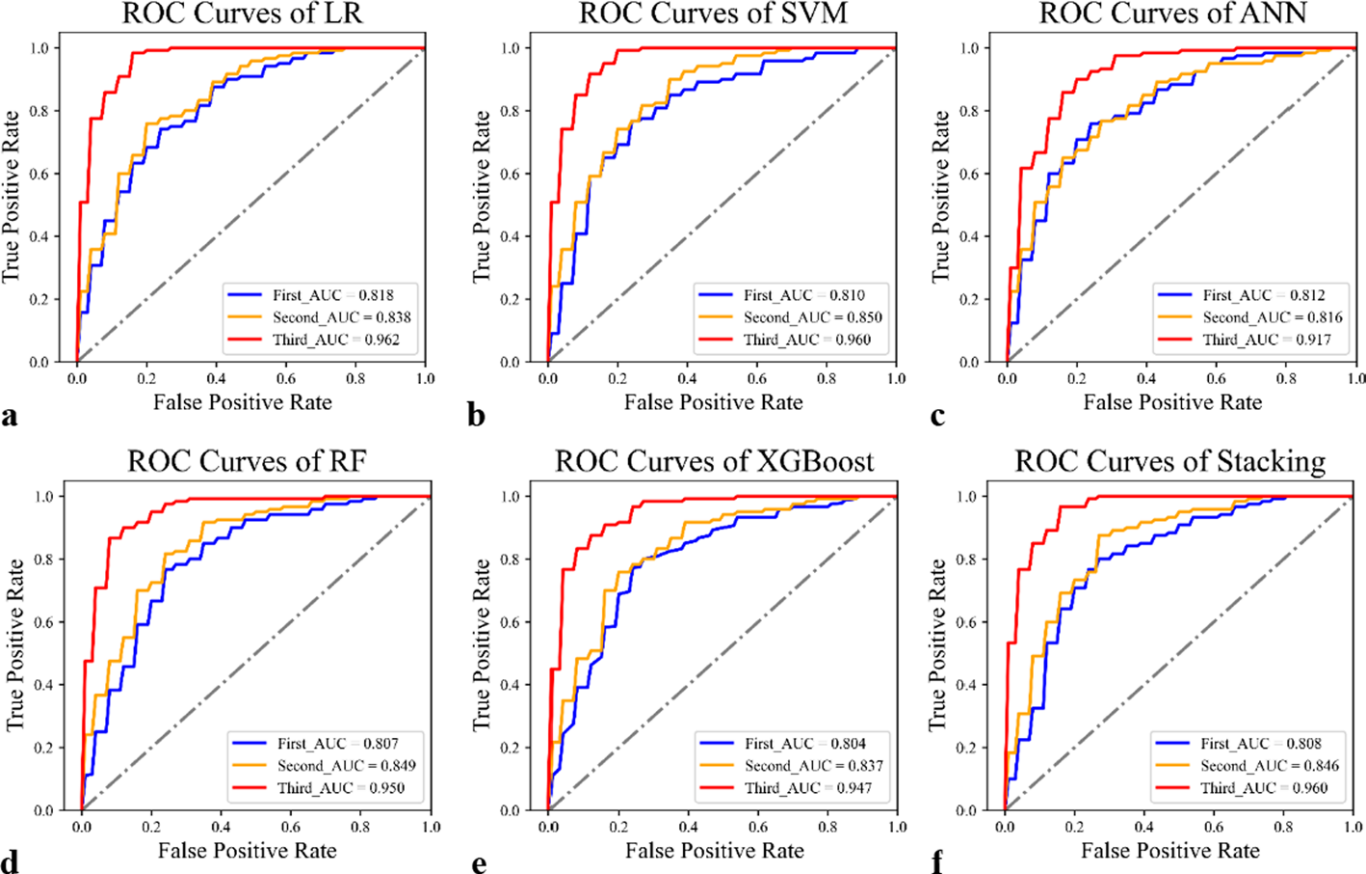
ROC curves of the proposed three-layer model based on **a** LR, **b** SVM, **c** ANN, **d** RF, **e** XGBoost and **f** stacking respectively on combination group

246 individuals who underwent both routine laboratory tests and CT scans were used to test the performances of three layers of the model. As shown in Table 3 and Fig. 4, more features used corresponded to better performance for all classifiers. Since the third layer used the most features, its performance was the best among the three layers. Among these classifiers, LR, showed the better performances than others. While, ANN performed worse especially in the second and third layer.

**Table 3:** Performance of proposed model based on each classifier

Layers		LR	SVM	ANN	RF	XGBoost	Stacking
1st Layer*	Training	0.848	0.849	0.823	0.899	0.897	0.883
	Test	**0.818**	0.810	0.812	0.807	0.804	0.808
2nd Layer*	Training	0.878	0.887	0.827	0.944	0.958	0.927
	Test	0.838	**0.850**	0.816	0.849	0.837	0.846
3rd Layer*	Training	0.983	0.980	0.909	0.995	0.993	0.989
	Test	**0.962**	0.960	0.917	0.950	0.947	0.960

The highest values among the six classifiers for each feature set in test set were highlighted in bold

*The first and second layer utilized demographic characteristics and clinical data respectively, while the third layer utilized clinical data and texture features

**Fig. 5:**
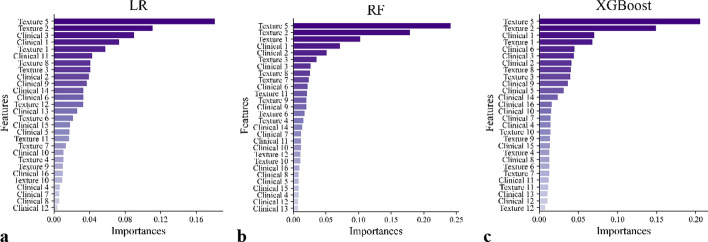
Feature importance for **a** LR, **b** RF and **c** XGBoost. These features were represented briefly by the combination of category and number. The details are listed in Additional file 1: Table S6

Figure 5 presents the feature importance for each classifier in discriminating individuals between osteoporosis and normal BMD. As demonstrated in Fig. 5, among the top 10 important features of each classifier, 8 features (3 clinical features and 5 texture features) were the same for three classifiers. To be specific, 3 clinical features referred to Clinical 1 (menopause status), Clinical 2 (age), and Clinical 3 (BMI). 5 texture features included Texture 1 (mean of GLCM’s mean contrast in 4 directions in ROIs), Texture 2 (mean of GLCM’s mean energy in 4 directions in ROIs), Texture 3 (mean of the standard deviation of GLCM’s homogeneity in 4 directions in ROIs), Texture 5 (mean of HI’s mean in ROIs) and Texture 8 (standard deviation of GLCM’s mean energy in 4 directions in ROIs). It can be found that HI’s mean denoted by Texture 5 was considered as the most important feature in three classifiers.

## Discussion

In this study, we proposed a hierarchical model as an alternative approach to differentiating individuals with and without osteoporosis. Considering the availability of the data, the hierarchical model was built with three layers utilizing demographic characteristics, clinical data, as well as clinical data and CT images, respectively. Six machine learning algorithms were used and LR, SVM and Stacking showed similar performances in the three layers according to Table 3. According to Occam's Razor that “entities should not be multiplied beyond necessity” [38], a simpler model based on LR would be preferred in our work, which achieved an AUC of 0.818, 0.838 and 0.962, respectively.

Clinical data have been used previously for the identification of individuals with osteoporosis or osteoporotic fracture [22, 23] and demographic characteristics, in particular, have been widely used in multiple osteoporosis assessment tools [18]. In the first and second layers of the proposed model, the performance of demographic characteristics and clinical data were tested, respectively. Demographic characteristics showed better performance than routine laboratory tests data but their combination slightly improved model performance for all classifiers as shown in Table 2. Moreover, all these classifiers provided similar performances, which demonstrated the effectiveness of clinical data in osteoporosis discrimination. Among clinical data, menopause status, age, and BMI were the most important indicators that were consistent with known risk factors [18, 22, 23]. Additionally, Clinical 6 (red blood cell count), Clinical 9 (alkaline phosphatase), and Clinical 11 (albumin) were also helpful in identifying individuals with osteoporosis, since they were considered as top 10 important features by one or two classifiers. What’s more, comparing to other osteoporosis assessment tools based on demographic characteristics only, the proposed model with an AUC of 0.818 showed better performance than OST (0.790) [14], ORAI (0.789) [15], and OSIRIS (0.710) [17], and similar performance with SCORE (0.811) [16]. However, it is should be noted that the performance (AUC: 0.827) of [20], which used 11 features, is better than our model based on 5 features. The reason is partly attributed to the more features used in [20].

It is difficult to distinguish differences in trabecular bone between osteoporosis and non-osteoporosis, even for experienced doctors [35], but texture analysis has been utilized in an attempt to solve the problem. Texture analysis is a non-invasive and quantitative image analysis method [24], which is used widely in medical images deriving from CT, X-ray, and MRI [39]. Several studies have suggested that texture analysis aided the discrimination of osteoporosis or osteoporotic fracture in multiple medical images [19]. In our study, several texture features deriving from GLCM, GLGM, and HI were extracted from CT images. Moreover, shape analysis was also employed to quantify the microarchitecture of trabecular bone, which probably could enhance the classification accuracy [29]. The results demonstrated that texture features were more important than shape features in detecting osteoporotic individuals, and the model performance was not improved utilizing both texture and shape features compared with that of texture features alone (Table 2). This could be partly explained by two reasons. On the one hand, shape features mainly described trabecular microarchitecture, while texture features reflected the condition of trabecular microarchitecture as well as cortical bone, containing more valuable information than shape features [24]. On the other hand, DXA test was used as the label to discriminate osteoporotic and normal individuals, however, DXA didn’t consider the influence of trabecular microarchitecture. As a result, conflicts may exist between shape features and model label. Based on this, shape features were not taken into account in the model.

As mentioned above, 5 texture features were of great importance in distinguishing patients with osteoporosis, including GLCM’s contrast (denoted by Texture 1), GLCM’s energy (denoted by Texture 2 and Texture 8), GLCM’s homogeneity (denoted by Texture 3), and HI’s mean (denoted by Texture 5). Contrast represents the depth of texture grooves and the image sharpness, energy reflects the orderliness and homogeneity computes the distribution compactness of GLCM diagonal elements [25]. Mean, the most important feature, reflects the mean signal intensity of images, which was consistent with other literature [35]. Meanwhile, violin plots shown in Additional file 1: Figure S1 demonstrated distributions of selected 47 features between two groups. As shown in Additional file 1: Figure S1, the five important texture features mentioned above exhibited the obvious differences between osteoporosis and non-osteoporosis. These features maybe reveal the distinct differences of cortical and cancellous bone between osteoporotic and non-osteoporotic individuals, such as the permeability of vertebral body increasing, cortical bone thinning, and trabecular bone disappearing in osteoporotic individuals [23].

**Fig. 6:**
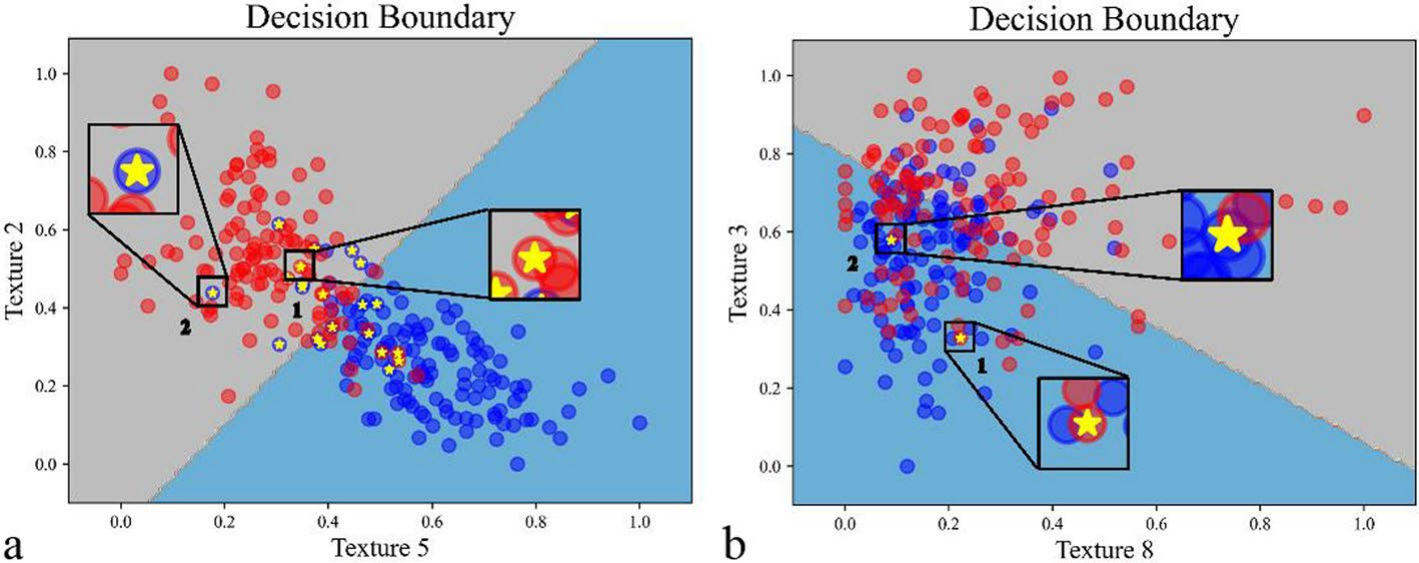
Decision boundary of LR based on Texture 5 and 2 (shown in **a**) as well as Texture 8 and 3 (shown in **b**). The gray and light blue area represent osteoporosis and normal individuals, respectively. Red and blue dots represent the samples labelled by osteoporosis and non-osteoporosis, respectively. Samples marked with yellow stars (such as dots marked with 1 and 2) represent the ones that are incorrectly classified by LR in five repeated experiments. All incorrectly classified samples are marked with yellow stars in (**a**), while only two are marked in (**b**)

Figure 6 showed the decision boundary of LR based on different predicting features (texture 5 and 2 as well as texture 8 and 3), which were important texture features mentioned above. The values of features in Fig. 6 were all scaled by Min–max normalization. As shown in Fig. 6a, most samples could be classified correctly, since the two most important features, texture 5 and 2 (shown in Fig. 5) were used. Samples marked with 1 and 2 were chosen to explain the underlying cause for wrongly classifications. Sample 1 with the label of osteoporosis was classified correctly according to texture 5 and 2 as shown in Fig. 6a, while it was classified wrongly based on texture 8 and 3 as shown in Fig. 6b. Similarly, predictions of sample 2 were inconsistent in Fig. 6a, b. Thus, it could be inferred that wrong classifications of the model were mainly due to confoundedness of predicting features.

We recognize that our study has some limitations. First, the number of samples was relatively small, especially for cases with CT images collected from a single center, which limited the robustness and generalization of the proposed model. Secondly, the image features were extracted from 2D CT slices, whilst 3D texture features are likely to contain more valuable information and have been used to screen osteoporotic fractures [26]. Adding 3D image features to our model is one of our on-going aims. Thirdly, lumbar vertebral bodies were segmented manually, which could lead to the variations of inter- and intra-observer on feature extraction. An automatic segmentation method is another question we will address in future work.

## Conclusions

The proposed model based on clinical data and CT images using machine learning methods showed great potential in opportunistic screening for osteoporosis without additional expense. In other words, different form DXA test, the features used in our model could be acquired for other purpose rather than osteoporosis detection only. Thus, it can be employed as an auxiliary tool for clinicians to screen whether an individual has a risk of osteoporosis in advance of a DXA test, which would be beneficial in scenarios without DXA equipment, e.g. community or family physical examination, and individuals with high osteoporosis risks but failing to take the DXA test. It is hoped that this model could serve to detect osteoporosis as early as possible and thereby prevent serious complications of osteoporosis, such as osteoporosis fractures.

## Supplementary Information

**Additional file 1**.** Table S1:** Distribution of participants’ clinical data between patients with osteoporosis and normal BMD. **Table S2:** Overview of the texture features extracted from CT images. **Table S3:** Overview of the shape features extracted from CT images. **Table S4:** Distribution of participants’ image features between patients with osteoporosis and normal BMD. **Table S5:** Detailed information of package used in python. **Table S6:** The selected features after statistical analysis. **Table S7:** The features used for three layers respectively. **Figure S1:** The violin plots of selected features after statistical analysis, including (**a**) clinical data, (**b**) texture features and (**c**) shape features. **Figure S2:** The correlation of selected (**a**) clinical data, (**b**) texture features and (**c**) shape features after statistical analysis in heatmap.

## Data Availability

Research data are not publicly available but can be obtained from the corresponding author on request after approval from the institutional review boards of all participating institutions.

## Abbreviations

BMD: Bone mineral density
DXA: Dual-energy X-ray absorptiometry
ROIs: Regions of interest
LR: Logistic regression
SVM: Support vector machine
ANN: Artificial neural network
RF: Random forests
XGBoost: EXtreme Gradient Boosting
ROC: Receiver operating characteristic
AUC: Area under the ROC curve
BMI: Body mass index
OST: Osteoporosis self-assessment tool
ORAI: Osteoporosis risk assessment instrument
SCORE: Simple calculated osteoporosis risk estimation
OSIRIS: Osteoporosis index of risk
ML: Machine learning
GLCM: Gray-Level Co-occurrence Matrix
GLGM: Gray-Level Gradient Matrix
HI: Histogram
