# Supplementary material for: A hierarchical opportunistic screening model for osteoporosis using machine learning applied to clinical data and CT images
Source: BMC Bioinformatics. 2022 Feb 10;23:63. doi: 10.1186/s12859-022-04596-z (PMC8829991; doi:10.1186/s12859-022-04596-z)
Supplement: Supplementary file 1 — Additional file 1. Table S1: Distribution of participants’ clinical data between patients with osteoporosis and normal BMD. Table S2: Overview of the texture features extracted from CT images. Table S3: Overview of the shape features extracted from CT images. Table S4: Distribution of participants’ image features between patients with osteoporosis and normal BMD. Table S5: Detailed information of package used in python. Table S6: The selected features after statistical analysis. Table S7: The features used for three layers respectively. Figure S1: The violin plots of selected features after statistical analysis, including (a) clinical data, (b) texture features and (c) shape features. Figure S2: The correlation of selected (a) clinical data, (b) texture features and (c) shape features after statistical analysis in heatmap. [file 12859_2022_4596_MOESM1_ESM.docx]

**Supplementary**

**Table S1** Distribution of participants’ clinical data bwtween patients with osteoporosis and normal BMD

| **Features** | **Total** | **Osteoporosis** | **Normal BMD** | ***P* value** |
| --- | --- | --- | --- | --- |
| No. of patients(%) | 2188(100.00) | 943(43.10) | 1245(56.90) |  |
| Menopause status, No.(%) |  |  |  | 0.000 |
| Premenopausal women | 290(13.25) | 8(0.85) | 282(22.65) |  |
| Postmenopausal women | 805(36.79) | 463(49.10) | 342(27.47) |  |
| Men | 1093(49.96) | 472(50.05) | 621(49.88) |  |
| Age, mean(SD), year | 60.04(11.05) | 65.67(9.69) | 55.77(10.06) | 0.000 |
| BMI, mean(SD), kg/m^2 | 24.98(3.39) | 24.22(3.39) | 25.56(3.27) | 0.000 |
| SP, mean(SD), mmHg | 133.67(19.07) | 136.02(18.87) | 131.88(19.03) | 0.000 |
| DP, mean(SD), mmHg | 78.57(12.02) | 78.17(12.33) | 78.88(11.77) | 0.869 |
| PP, mean(SD), mmHg | 55.09(14.93) | 57.85(15.2) | 53.00(14.38) | 0.000 |
| WBC, mean(SD), 10^9/L | 6.20(1.82) | 6.28(1.99) | 6.15(1.67) | 0.113 |
| RNEU, mean(SD), % | 57.91(9.23) | 58.14(9.49) | 57.74(9.02) | 0.395 |
| RLYM, mean(SD), % | 32.07(8.55) | 31.95(9.04) | 32.16(8.16) | 0.428 |
| RMON, mean(SD), % | 7.23(2.15) | 7.2(2.09) | 7.25(2.2) | 0.530 |
| ANEU, mean(SD), 10^9/L | 3.65(1.37) | 3.69(1.37) | 3.62(1.37) | 0.202 |
| ALYM, mean(SD), 10^9/L | 1.95(0.88) | 1.99(1.17) | 1.93(0.57) | 0.789 |
| AMON, mean(SD), 10^9/L | 0.44(0.16) | 0.44(0.16) | 0.44(0.16) | 0.088 |
| RBC, mean(SD), 10^12/L | 4.62(0.51) | 4.57(0.53) | 4.66(0.48) | 0.000 |
| HGB, mean(SD), g/L | 139.97(16.74) | 139.13(16.3) | 140.61(17.04) | 0.005 |
| HCT, mean(SD) | 42.48(4.69) | 42.34(4.8) | 42.58(4.59) | 0.087 |
| PLT, mean(SD), 10^9/L | 242.3(64.78) | 238.67(67.08) | 245.05(62.84) | 0.000 |
| PCT, mean(SD), % | 0.25(0.06) | 0.24(0.06) | 0.25(0.06) | 0.000 |
| ALT, mean(SD), U/L | 20.05(15.98) | 18.85(13.89) | 20.96(17.34) | 0.000 |
| AST, mean(SD), U/L | 21.76(11.01) | 22.05(13.3) | 21.53(8.89) | 0.172 |
| GGT, mean(SD), U/L | 28.38(27.8) | 28.1(26.35) | 28.58(28.85) | 0.418 |
| AKP, mean(SD), U/L | 78.79(25.32) | 85.56(26.03) | 73.67(23.51) | 0.000 |
| TBIL, mean(SD), μmol/L | 13.34(6.65) | 13.57(7.33) | 13.17(6.07) | 0.153 |
| DBIL, mean(SD), μmol/L | 3.27(1.49) | 3.3(1.45) | 3.25(1.51) | 0.052 |
| IBIL, mean(SD), μmol/L | 10.07(5.7) | 10.27(6.44) | 9.92(5.07) | 0.360 |
| TP, mean(SD), g/L | 71.0(5.59) | 70.72(6.07) | 71.21(5.18) | 0.037 |
| ALB, mean(SD), g/L | 44.02(3.39) | 43.61(3.43) | 44.33(3.32) | 0.000 |
| GLB, mean(SD), g/L | 27.05(4.25) | 27.24(4.32) | 26.91(4.2) | 0.033 |
| Cho, mean(SD), mmol/L | 4.94(1.02) | 5.0(1.04) | 4.89(1.01) | 0.000 |
| HDL-C, mean(SD), mmol/L | 1.33(0.32) | 1.36(0.32) | 1.32(0.32) | 0.000 |
| LDL-C, mean(SD), mmol/L | 2.98(0.77) | 3.0(0.79) | 2.96(0.76) | 0.006 |
| TG, mean(SD), mmol/L | 1.49(0.95) | 1.48(1.03) | 1.49(0.88) | 0.945 |
| GLU, mean(SD), mmol/L | 5.59(1.43) | 5.67(1.41) | 5.53(1.44) | 0.000 |
| BUN, mean(SD), mmol/L | 5.33(1.72) | 5.43(1.82) | 5.26(1.63) | 0.003 |
| Cr, mean(SD), μmol/L | 67.26(18.4) | 67.15(17.79) | 67.34(18.84) | 0.795 |

Abbreviations: *N* number of features; *MPS* menopause status; *SP* systolic pressure; *DP* diastolic pressure; *PP* pulse pressure; *WBC* white blood cell count; *RNEU* relative neutrophil count; *RLYM* relative lymphocyte count; *RMON* relative monocyte count; *ANEU* absolute neutrophil count; *ALYM* absolute lymphocyte count; *AMON* absolute monocyte count; *RBC* red blood cell count; *HGB* haemoglobin; *HCT* haematocrit; *PLT* platelet count; *PCT* plateletcrit; *ALT* alanine transaminase; *AST* aspartate transaminase; *GGT* $\gamma$-glutamyl transpeptidase; *AKP* alkaline phosphatase; *TBIL* total bilirubin; *DBIL* direct bilirubin; *IBIL* indirect bilirubin; *TP* total protein; *ALB* albumin, GLB: globulin; *Cho* total cholesterol; *HDL-C* high-density lipoprotein cholesterol; *LDL-C* low-density lipoprotein cholesterol; *TG* triglyceride; *GLU* glucose; *BUN* blood urea nitrogen; *Cr* creatinine

**Table S2** Overview of the texture features extracted from CT images

| **Texture Parameters** | | **Features** | **Physical meaning** | **N** |
| --- | --- | --- | --- | --- |
| GLCM | contrast (Con), correlation (Cor), energy (Ene), homogeneity (Hg),  entropy (Ent) | Mean_GLCM_Mean_Con, Mean_GLCM_Std_Con, Mean_GLCM_Mean_Cor, Mean_GLCM_Std_Cor,  Mean_GLCM_Mean_Ene, Mean_GLCM_Std_Ene,  Mean_GLCM_Mean_Hg, Mean_GLCM_Std_Hg,  Mean_GLCM_Mean_Ent, Mean_GLCM_Std_Ent | the mean of mean and standard deviation of each GLCM parameter in four directions in ROIs | 20 |
|  |  | Std_GLCM_Mean_Con, Std_GLCM_Std_Con Std_GLCM_Mean_Cor, Std_GLCM_Std_Cor  Std_GLCM_Mean_Ene, Std_GLCM_Std_Ene  Std_GLCM_Mean_Hg, Std_GLCM_Std_Hg Std_GLCM_Mean_Ent, Std_GLCM_Std_Ent | the standard deviation of mean and standard deviation of each GLCM parameter in four directions in ROIs |  |
| GLGM | mean,  variance (Var), skewness (Ske), kurtosis (Kur) | Mean_GLGM_Mean, Mean_GLGM_Var,  Mean_GLGM_Ske, Mean_GLGM_Kur, | the mean of each GLGM parameter in ROIs | 8 |
|  |  | Std_GLGM_Mean, Std_GLGM_Var, Std_GLGM_Ske, Std_GLGM_Kur | the standard deviation of each GLGM parameter in ROIs |  |
| HI | mean, variance (Var), skewness (Ske), kurtosis (Kur), energy (Ene), entropy (Ent) | Mean_HI_Mean, Mean_HI_Var, Mean_HI_Ske, Mean_HI_Kur, Mean_HI_Ene, Mean_HI_Ent | the mean of each HI parameter in ROIs | 12 |
|  |  | Std_HI_Mean, Std_HI_Var,  Std_HI_Ske, Std_HI_Kur,  Std_HI_Ene, Std_HI_Ent | The standard deviation of each HI parameter in ROIs |  |

Abbreviations: *GLCM* Gray-Level Co-occurrence Matrix; *GLGM* Gray-Level Gradient Matrix; *HI* Gray-Level Histogram

**Table S3** Overview of the shape features extracted from CT images

| **Shape Parameters** | **Features** | **Physical meaning** | **N** |
| --- | --- | --- | --- |
| perimeter (Pm), area,  regional density (Rd),  length-width ratio (Lwr),  circularity (Cir),  solidity (Sol), rectangularity (Rec),  Hu’s invariant moments  (Hu1-Hu7) | Mean_Shape_Mean_Pm, Mean_Shape_Std_Pm,  Mean_Shape_Mean_Area, Mean_Shape_Std_Area,  Mean_Shape_Mean_Rd, Mean_Shape_Std_Rd,  Mean_Shape_Mean_Lwr, Mean_Shape_Std_Lwr,  Mean_Shape_Mean_Cir, Mean_Shape_Std_Cir,  Mean_Shape_Mean_Sol, Mean_Shape_Std_Sol,  Mean_Shape_Mean_Rec, Mean_Shape_Std_Rec,  Mean_Shape_Mean_Hu1, Mean_Shape_Std_Hu1,  Mean_Shape_Std_Hu2, Mean_Shape_Mean_Hu2,  Mean_Shape_Std_Hu3, Mean_Shape_Mean_Hu3,  Mean_Shape_Mean_Hu4, Mean_Shape_Std_Hu4,  Mean_Shape_Mean_Hu5, Mean_Shape_Std_Hu5,  Mean_Shape_Mean_Hu6, Mean_Shape_Std_Hu6,  Mean_Shape_Mean_Hu7, Mean_Shape_Std_Hu7 | the mean of mean and standard deviation of each shape parameter in segmented regions in ROIs | 28 |
|  | Std_Shape_Mean_Pm, Std_Shape_Std_Pm,  Std_Shape_Mean_Area, Std_Shape_Std_Area,  Std_Shape_Mean_Rd, Std_Shape_Std_Rd,  Std_Shape_Mean_Lwr, Std_Shape_Std_Lwr,  Std_Shape_Mean_Cir, Std_Shape_Std_Cir,  Std_Shape_Mean_Sol, Std_Shape_Std_Sol,  Std_Shape_Mean_Rec, Std_Shape_Std_Rec,  Std_Shape_Mean_Hu1, Std_Shape_Std_Hu1,  Std_Shape_Std_Hu2, Std_Shape_Mean_Hu2,  Std_Shape_Std_Hu3, Std_Shape_Mean_Hu3,  Std_Shape_Mean_Hu4, Std_Shape_Std_Hu4,  Std_Shape_Mean_Hu5, Std_Shape_Std_Hu5,  Std_Shape_Mean_Hu6, Std_Shape_Std_Hu6,  Std_Shape_Mean_Hu7, Std_Shape_Std_Hu7 | the standard deviation of mean and standard deviation of each shape parameter in segmented regions in ROIs | 28 |

**Table S4** Distribution of participants’ image features between patients with osteoporosis and normal BMD

| **Features** | **Total** | **Osteoporosis** | **Normal BMD** | ***P* value** |
| --- | --- | --- | --- | --- |
| No. of patients(%) | 268(100.00) | 126(47.01) | 142(52.99) |  |
| Mean_GLCM_Mean_CON, mean(SD) | 87.7(34.03) | 67.34(19.81) | 105.77(33.81) | 0.000 |
| Mean_GLCM_Std_CON, mean(SD) | 34.31(13.52) | 26.28(8.05) | 41.44(13.4) | 0.000 |
| Mean_GLCM_Mean_COR, mean(SD) | 0.9(0.01) | 0.9(0.01) | 0.9(0.01) | 0.951 |
| Mean_GLCM_Std_COR, mean(SD) | 0.04(0.01) | 0.04(0.01) | 0.04(0.0) | 0.244 |
| Mean_GLCM_Mean_ASM, mean(SD) | 0.0(0.0) | 0.0(0.0) | 0.0(0.0) | 0.000 |
| Mean_GLCM_Std_ASM, mean(SD) | 0.0(0.0) | 0.0(0.0) | 0.0(0.0) | 0.000 |
| Mean_GLCM_Mean_IDM, mean(SD) | 0.31(0.02) | 0.32(0.02) | 0.3(0.02) | 0.000 |
| Mean_GLCM_Std_IDM, mean(SD) | 0.05(0.01) | 0.06(0.01) | 0.05(0.01) | 0.000 |
| Mean_GLCM_Mean_ENT, mean(SD) | 2.99(0.09) | 2.92(0.07) | 3.05(0.07) | 0.000 |
| Mean_GLCM_Std_ENT, mean(SD) | 0.08(0.01) | 0.09(0.01) | 0.07(0.01) | 0.000 |
| Mean_GGCM_Mean, mean(SD) | 13.65(2.25) | 12.2(1.52) | 14.95(1.99) | 0.000 |
| Mean_GGCM_Var, mean(SD) | 190.87(83.51) | 141.47(50.27) | 234.7(82.66) | 0.000 |
| Mean_GGCM_Ske, mean(SD) | 2.02(0.25) | 2.03(0.29) | 2.01(0.2) | 0.744 |
| Mean_GGCM_Kur, mean(SD) | 5.04(1.7) | 5.37(2.02) | 4.75(1.28) | 0.013 |
| Mean_HI_Mean, mean(SD) | 101.35(7.57) | 95.23(4.28) | 106.77(5.4) | 0.000 |
| Mean_HI_Var, mean(SD) | 454.96(174.38) | 348.55(109.44) | 549.37(166.73) | 0.000 |
| Mean_HI_Ske, mean(SD) | 1.38(0.4) | 1.46(0.4) | 1.3(0.39) | 0.000 |
| Mean_HI_Kur, mean(SD) | 6.51(1.59) | 6.87(1.71) | 6.18(1.41) | 0.000 |
| Mean_HI_Ene, mean(SD) | 0.02(0.0) | 0.02(0.0) | 0.02(0.0) | 0.000 |
| Mean_HI_Ent, mean(SD) | 4.21(0.18) | 4.08(0.12) | 4.33(0.13) | 0.000 |
| Std_GLCM_Mean_CON, mean(SD) | 15.66(10.98) | 12.83(9.84) | 18.17(11.33) | 0.000 |
| Std_GLCM_Std_CON, mean(SD) | 6.81(5.09) | 5.63(4.47) | 7.85(5.37) | 0.000 |
| Std_GLCM_Mean_COR, mean(SD) | 0.01(0.01) | 0.01(0.01) | 0.01(0.01) | 0.481 |
| Std_GLCM_Std_COR, mean(SD) | 0.0(0.0) | 0.0(0.0) | 0.0(0.0) | 0.596 |
| Std_GLCM_Mean_ASM, mean(SD) | 0.0(0.0) | 0.0(0.0) | 0.0(0.0) | 0.000 |
| Std_GLCM_Std_ASM, mean(SD) | 0.0(0.0) | 0.0(0.0) | 0.0(0.0) | 0.000 |
| Std_GLCM_Mean_IDM, mean(SD) | 0.01(0.01) | 0.01(0.01) | 0.01(0.01) | 0.223 |
| Std_GLCM_Std_IDM, mean(SD) | 0.0(0.0) | 0.0(0.0) | 0.0(0.0) | 0.962 |
| Std_GLCM_Mean_ENT, mean(SD) | 0.04(0.03) | 0.04(0.03) | 0.04(0.02) | 0.027 |
| Std_GLCM_Std_ENT, mean(SD) | 0.01(0.0) | 0.01(0.0) | 0.01(0.0) | 0.496 |
| Std_GGCM_Mean, mean(SD) | 1.05(0.68) | 0.97(0.68) | 1.13(0.67) | 0.016 |
| Std_GGCM_Var, mean(SD) | 40.22(29.28) | 32.69(25.95) | 46.89(30.42) | 0.000 |
| Std_GGCM_Ske, mean(SD) | 0.24(0.17) | 0.28(0.2) | 0.22(0.13) | 0.005 |
| Std_GGCM_Kur, mean(SD) | 1.68(1.45) | 1.98(1.78) | 1.41(1.0) | 0.001 |
| Std_HI_Mean, mean(SD) | 1.92(1.36) | 1.57(1.21) | 2.24(1.41) | 0.000 |
| Std_HI_Var, mean(SD) | 92.47(75.73) | 76.56(60.98) | 106.6(84.27) | 0.000 |
| Std_HI_Ske, mean(SD) | 0.3(0.19) | 0.29(0.23) | 0.3(0.16) | 0.094 |
| Std_HI_Kur, mean(SD) | 1.23(0.9) | 1.39(1.08) | 1.09(0.68) | 0.026 |
| Std_HI_Ene, mean(SD) | 0.0(0.0) | 0.0(0.0) | 0.0(0.0) | 0.001 |
| Std_HI_Ent, mean(SD) | 0.08(0.05) | 0.09(0.05) | 0.08(0.06) | 0.110 |
| Mean_Shape_Mean_Perimeter, mean(SD) | 123.65(44.38) | 104.41(30.2) | 140.72(47.84) | 0.000 |
| Mean_Shape_Std_Perimeter, mean(SD) | 125.64(41.45) | 113.19(33.64) | 136.69(44.49) | 0.000 |
| Mean_Shape_Mean_Area, mean(SD) | 348.86(122.91) | 307.31(92.21) | 385.73(134.44) | 0.000 |
| Mean_Shape_Std_Area, mean(SD) | 448.35(127.82) | 441.57(120.34) | 454.37(133.82) | 0.190 |
| Mean_Shape_Mean_Density, mean(SD) | 0.04(0.01) | 0.04(0.01) | 0.04(0.01) | 0.000 |
| Mean_Shape_Std_Density, mean(SD) | 0.03(0.01) | 0.03(0.01) | 0.03(0.01) | 0.001 |
| Mean_Shape_Mean_Ratio, mean(SD) | 2.83(0.74) | 2.97(0.52) | 2.71(0.88) | 0.000 |
| Mean_Shape_Std_Ratio, mean(SD) | 2.14(0.88) | 2.34(0.67) | 1.97(1.0) | 0.000 |
| Mean_Shape_Mean_Circularity, mean(SD) | 0.51(0.12) | 0.55(0.11) | 0.46(0.11) | 0.000 |
| Mean_Shape_Std_Circularity, mean(SD) | 0.39(0.1) | 0.41(0.09) | 0.37(0.1) | 0.001 |
| Mean_Shape_Mean_Solidity, mean(SD) | 0.73(0.07) | 0.76(0.05) | 0.69(0.06) | 0.000 |
| Mean_Shape_Std_Solidity, mean(SD) | 0.17(0.05) | 0.14(0.04) | 0.2(0.04) | 0.000 |
| Mean_Shape_Mean_rectangle_ratio, mean(SD) | 0.56(0.06) | 0.59(0.05) | 0.53(0.05) | 0.000 |
| Mean_Shape_Std_rectangle_ratio, mean(SD) | 0.15(0.03) | 0.13(0.03) | 0.16(0.03) | 0.000 |
| Mean_Shape_Mean_Hu1, mean(SD) | 0.45(0.09) | 0.42(0.07) | 0.48(0.09) | 0.000 |
| Mean_Shape_Mean_Hu2, mean(SD) | 0.19(0.11) | 0.19(0.09) | 0.2(0.13) | 0.678 |
| Mean_Shape_Mean_Hu3, mean(SD) | 0.05(0.06) | 0.02(0.03) | 0.06(0.07) | 0.000 |
| Mean_Shape_Mean_Hu4, mean(SD) | 0.01(0.02) | 0.01(0.01) | 0.02(0.02) | 0.000 |
| Mean_Shape_Mean_Hu5, mean(SD) | 0.0(0.03) | 0.0(0.01) | 0.0(0.04) | 0.000 |
| Mean_Shape_Mean_Hu6, mean(SD) | 0.01(0.02) | 0.01(0.01) | 0.0(0.02) | 0.001 |
| Mean_Shape_Mean_Hu7, mean(SD) | 0.0(0.06) | 0.0(0.0) | 0.0(0.09) | 0.768 |
| Mean_Shape_Std_Hu1, mean(SD) | 0.29(0.09) | 0.26(0.08) | 0.31(0.09) | 0.000 |
| Mean_Shape_Std_Hu2, mean(SD) | 0.28(0.17) | 0.29(0.16) | 0.27(0.19) | 0.071 |
| Mean_Shape_Std_Hu3, mean(SD) | 0.09(0.12) | 0.05(0.06) | 0.12(0.14) | 0.000 |
| Mean_Shape_Std_Hu4, mean(SD) | 0.03(0.03) | 0.02(0.03) | 0.03(0.03) | 0.000 |
| Mean_Shape_Std_Hu5, mean(SD) | 0.02(0.07) | 0.01(0.03) | 0.04(0.08) | 0.000 |
| Mean_Shape_Std_Hu6, mean(SD) | 0.02(0.04) | 0.02(0.04) | 0.02(0.03) | 0.059 |
| Mean_Shape_Std_Hu7, mean(SD) | 0.03(0.11) | 0.0(0.01) | 0.05(0.15) | 0.000 |
| Std_Shape_Mean_Perimeter, mean(SD) | 37.96(36.97) | 28.25(23.88) | 46.58(43.76) | 0.000 |
| Std_Shape_Std_Perimeter, mean(SD) | 43.71(33.02) | 32.2(24.74) | 53.93(35.96) | 0.000 |
| Std_Shape_Mean_Area, mean(SD) | 106.08(106.93) | 85.07(78.08) | 124.71(124.22) | 0.000 |
| Std_Shape_Std_Area, mean(SD) | 127.26(92.8) | 109.29(79.18) | 143.2(100.75) | 0.004 |
| Std_Shape_Mean_Density, mean(SD) | 0.01(0.01) | 0.01(0.01) | 0.01(0.01) | 0.014 |
| Std_Shape_Std_Density, mean(SD) | 0.01(0.01) | 0.01(0.01) | 0.01(0.01) | 0.000 |
| Std_Shape_Mean_Ratio, mean(SD) | 0.74(0.55) | 0.64(0.42) | 0.83(0.62) | 0.039 |
| Std_Shape_Std_Ratio, mean(SD) | 0.89(0.65) | 0.75(0.48) | 1.02(0.75) | 0.006 |
| Std_Shape_Mean_Circularity, mean(SD) | 0.14(0.07) | 0.12(0.07) | 0.15(0.07) | 0.014 |
| Std_Shape_Std_Circularity, mean(SD) | 0.14(0.07) | 0.12(0.06) | 0.16(0.07) | 0.000 |
| Std_Shape_Mean_Solidity, mean(SD) | 0.06(0.03) | 0.05(0.03) | 0.06(0.03) | 0.000 |
| Std_Shape_Std_Solidity, mean(SD) | 0.04(0.02) | 0.04(0.02) | 0.05(0.02) | 0.001 |
| Std_Shape_Mean_rectangle_ratio, mean(SD) | 0.05(0.03) | 0.05(0.03) | 0.06(0.03) | 0.006 |
| Std_Shape_Std_rectangle_ratio, mean(SD) | 0.04(0.02) | 0.04(0.02) | 0.04(0.02) | 0.064 |
| Std_Shape_Mean_Hu1, mean(SD) | 0.08(0.06) | 0.07(0.04) | 0.1(0.06) | 0.000 |
| Std_Shape_Mean_Hu2, mean(SD) | 0.11(0.11) | 0.09(0.07) | 0.13(0.13) | 0.002 |
| Std_Shape_Mean_Hu3, mean(SD) | 0.05(0.07) | 0.02(0.04) | 0.07(0.09) | 0.000 |
| Std_Shape_Mean_Hu4, mean(SD) | 0.01(0.02) | 0.01(0.01) | 0.02(0.02) | 0.000 |
| Std_Shape_Mean_Hu5, mean(SD) | 0.02(0.05) | 0.01(0.02) | 0.03(0.07) | 0.000 |
| Std_Shape_Mean_Hu6, mean(SD) | 0.01(0.03) | 0.01(0.02) | 0.01(0.03) | 0.005 |
| Std_Shape_Mean_Hu7, mean(SD) | 0.02(0.09) | 0.0(0.01) | 0.03(0.12) | 0.000 |
| Std_Shape_Std_Hu1, mean(SD) | 0.09(0.06) | 0.08(0.06) | 0.1(0.07) | 0.029 |
| Std_Shape_Std_Hu2, mean(SD) | 0.18(0.19) | 0.16(0.14) | 0.2(0.21) | 0.060 |
| Std_Shape_Std_Hu3, mean(SD) | 0.1(0.14) | 0.05(0.08) | 0.14(0.17) | 0.000 |
| Std_Shape_Std_Hu4, mean(SD), mean(SD) | 0.03(0.04) | 0.02(0.03) | 0.03(0.04) | 0.000 |
| Std_Shape_Std_Hu5, mean(SD), mean(SD) | 0.04(0.1) | 0.01(0.05) | 0.05(0.13) | 0.000 |
| Std_Shape_Std_Hu6, mean(SD), mean(SD) | 0.03(0.06) | 0.03(0.06) | 0.03(0.06) | 0.018 |
| Std_Shape_Std_Hu7, mean(SD), mean(SD) | 0.04(0.17) | 0.01(0.02) | 0.07(0.22) | 0.000 |

**Table S5** Detailed information of package used in python

| **Package_name** | **Version** |
| --- | --- |
| numpy | 1.17.4+mkl |
| pandas | 0.25.3 |
| scikit-learn | 0.24.1 |
| matplotlib | 3.2.0rc2 |
| xgboost | 1.3.3 |

**Table S6** The selected features after statistical analysis

|  | **Features** | **N** |
| --- | --- | --- |
| Demographic Characteristics | MPS, age, BMI, SP, PP | 5 |
| Routine Laboratory Tests | RBC, PLT, ALT, AKP, TP, ALB, GLB, Cho, HDL-C, GLU, BUN | 11 |
| Texture Features | Mean_GLCM_Mean_Con, Mean_GLCM_Mean_Ene, Mean_GLCM_Std_Hg, Mean_GLGM_Kur, Mean_HI_Mean, Mean_HI_Ske, Std_GLCM_Mean_Con, Std_GLCM_Mean_Ene, Std_GLCM_Std_Ene, Std_GLGM_Ske, Std_HI_Mean, Std_HI_Kur | 12 |
| Shape Features | Mean_Shape_Mean_Pm, Mean_Shape_Std_Pm, Mean_Shape_Mean_Rd, Mean_Shape_Std_Rd, Mean_Shape_Mean_Sol, Mean_Shape_Std_Sol, Mean_Shape_Std_Rec, Mean_Shape_Mean_Hu3, Mean_Shape_Mean_Hu5, Std_Shape_Mean_Pm, Std_Shape_Std_Pm, Std_Shape_Std_Area, Std_Shape_Mean_Rd, Std_Shape_Std_Rd, Std_Shape_Mean_Lwr, Std_Shape_Mean_Sol, Std_Shape_Std_Sol, Std_Shape_Mean_Hu1, Std_Shape_Std_Hu1 | 19 |

The order of these features is consisted with Fig. 5, i.e. Clinical 1 = MPS, Clinical 2 = age, Clinical 3 = BMI, Clinical 4 = SP, Clinical 5 = PP, Clinical 6 = RBC, Clinical 7 = PLT, Clinical 8 = ALT, Clinical 9 = AKP, Clinical 10 = TP, Clinical 11 = ALB, Clinical 12 = GLB, Clinical 13 = Cho, Clinical 14 = HDL-C, Clinical 15 = GLU, Clinical 16 = BUN, Texture 1 = Mean_ GLCM_Mean_Con, Texture 2 = Mean_GLCM_Mean_Ene, Texture 3 = Mean_GLCM_Std_Hg, Texture 4 = Mean_GLGM_ Kur, Texture 5 = Mean_HI_Mean, Texture 6 = Mean_HI_Ske, Texture 7 = Std_GLCM_Mean_Con, Texture 8 = Std_GLCM_ Mean_Ene, Texture 9 = Std_GLCM_Std_Ene, Texture 10 = Std_GLGM_Ske, Texture 11 = Std_HI_Mean, Texture 12 = Std_ HI_Kur, Shape 1 = Mean_Shape_Mean_Pm, Shape 2 = Mean_Shape_Std_Pm, Shape 3 = Mean_Shape_Mean_Rd, Shape 4 = Mean_Shape_Std_Rd, Shape 5 = Mean_Shape_Mean_Sol, Shape 6 = Mean_Shape_Std_Sol, Shape 7 = Mean_Shape_Std_ Rec, Shape 8 = Mean_Shape_Mean_Hu3, Shape 9 = Mean_Shape_Mean_Hu5, Shape 10 = Std_Shape_Mean_Pm, Shape 11 = Std_Shape_Std_Pm, Shape 12 = Std_Shape_Std_Area, Shape 13 = Std_Shape_Mean_Rd, Shape 14 = Std_Shape_Std_Rd, Shape 15 = Std_Shape_Mean_Lwr, Shape 16 = Std_Shape_Mean_Sol, Shape 17 = Std_Shape_Std_Sol, Shape 18 = Std_ Shape_Mean_Hu1, Shape 19 = Std_Shape_Std_Hu1

**Table S7** The features used for three layers respectively

|  | **Features** | **N** |
| --- | --- | --- |
| 1^st^ Layer | MPS, age, BMI, SP, PP | 5 |
| 2^nd^ Layer | MPS, age, BMI, SP, PP, RBC, PLT, ALT, AKP, TP, ALB, GLB, Cho, HDL-C, GLU, BUN | 16 |
| 3^rd^ Layer | MPS, age, BMI, SP, PP, RBC, PLT, ALT, AKP, TP, ALB, GLB, Cho, HDL-C, GLU, BUN, Mean_GLCM_Mean_Con, Mean_GLCM_Mean_Ene, Mean_GLCM_Std_Hg, Mean_GLGM_Kur, Mean_HI_Mean, Mean_HI_Ske, Std_GLCM_Mean_Con, Std_GLCM_Mean_Ene, Std_GLCM_Std_Ene, Std_GLGM_Ske, Std_HI_Mean, Std_HI_Kur | 28 |

1.
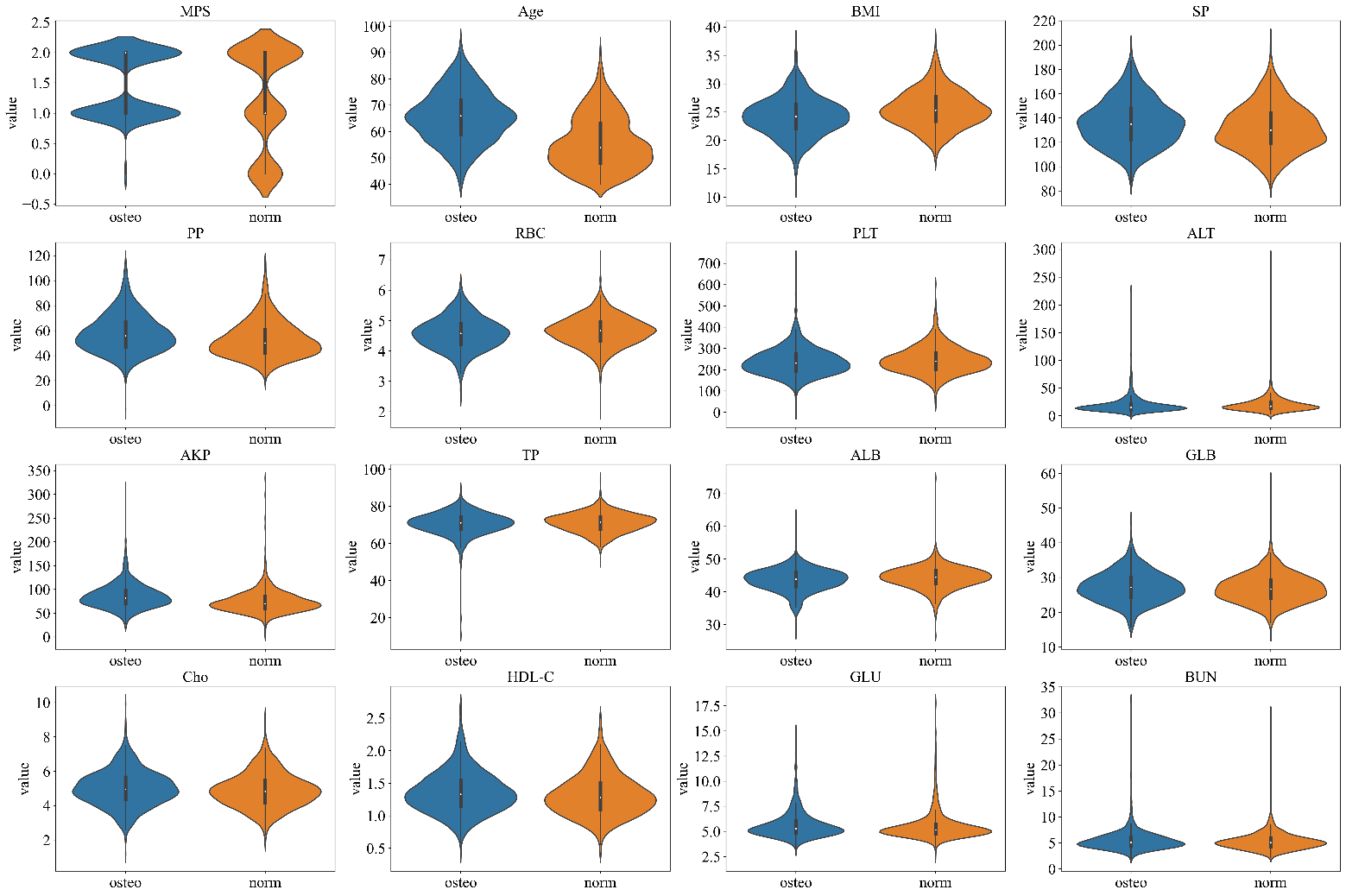


**b.
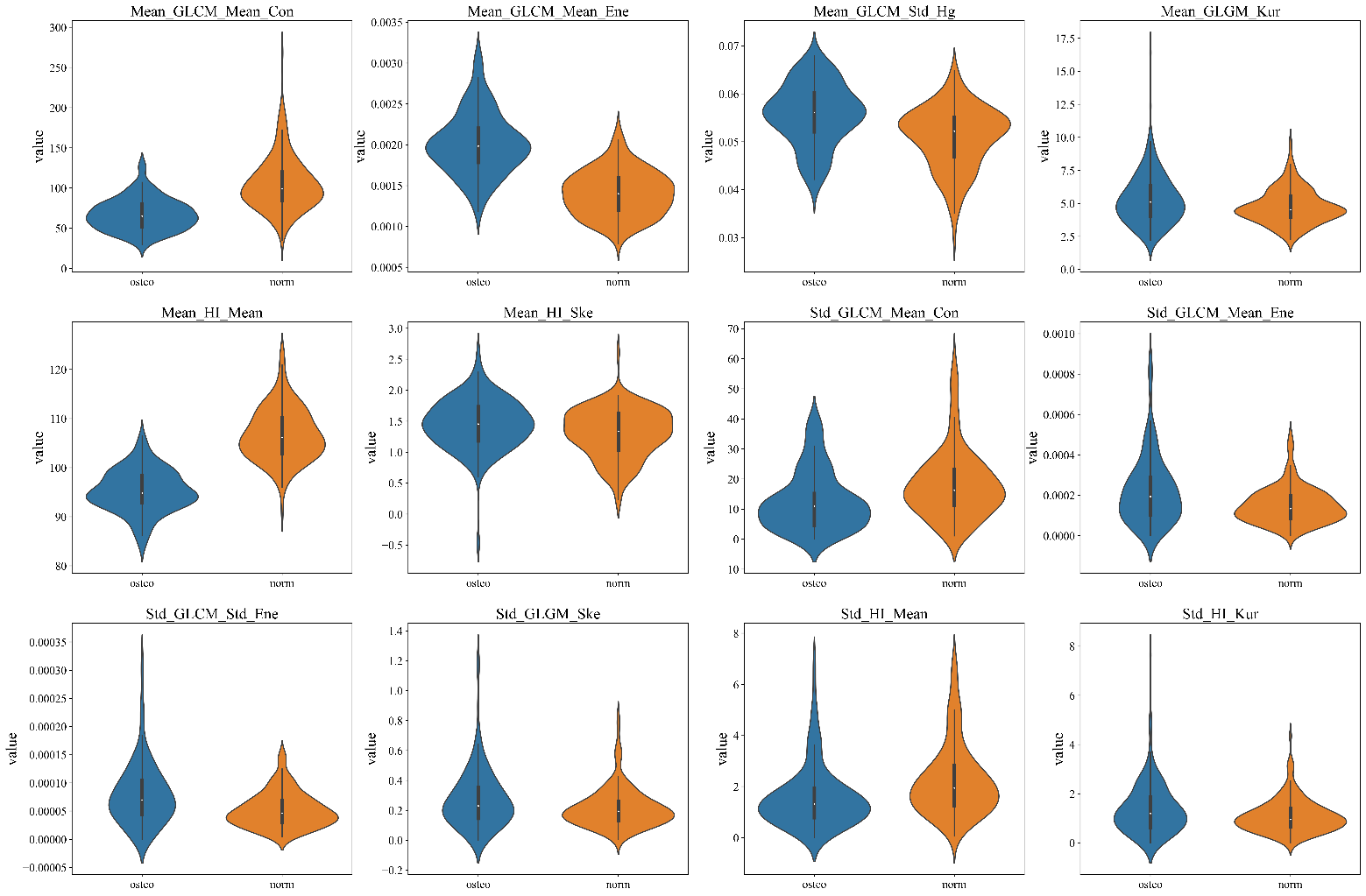
**

**c.
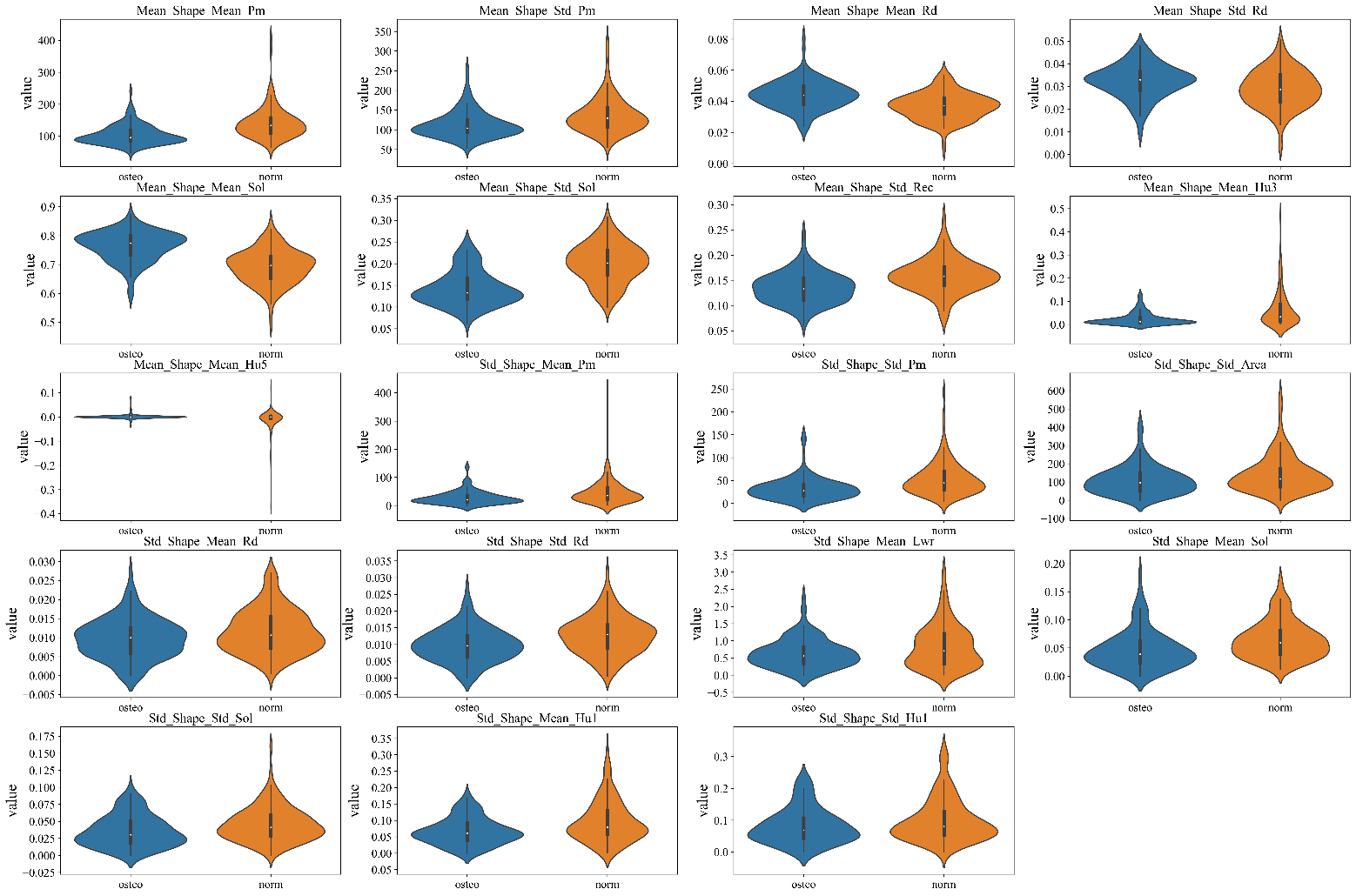
**

**Figure S1** The violin plots of selected features after statistical analysis, including (**a**) clinical data, (**b**) texture features and (**c**) shape features. For MPS shown in (a), 0, 1, 2 represents women not in menopause status, women in menopause status and men.

**a.**
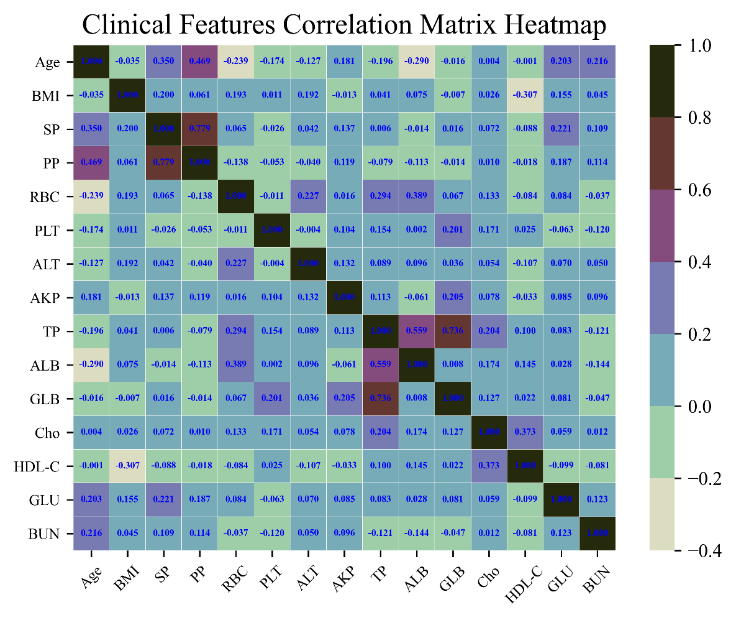
 **b.
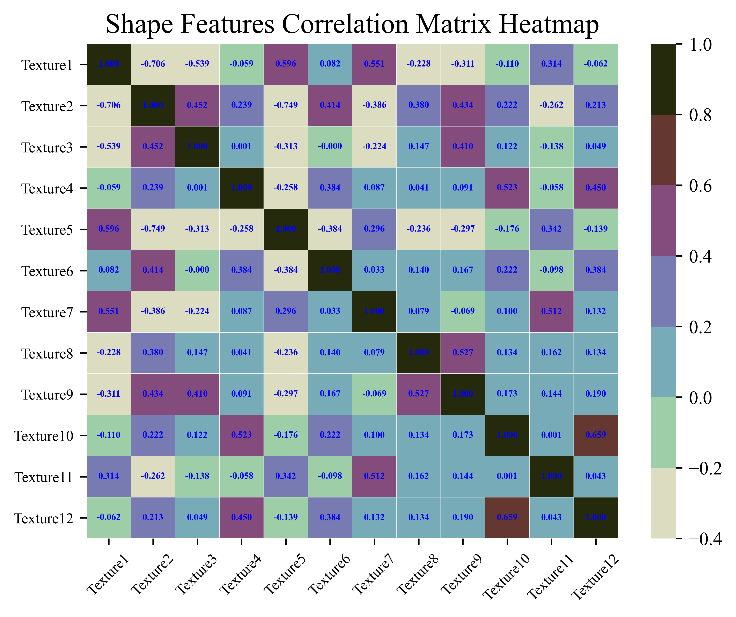
**

**c.**
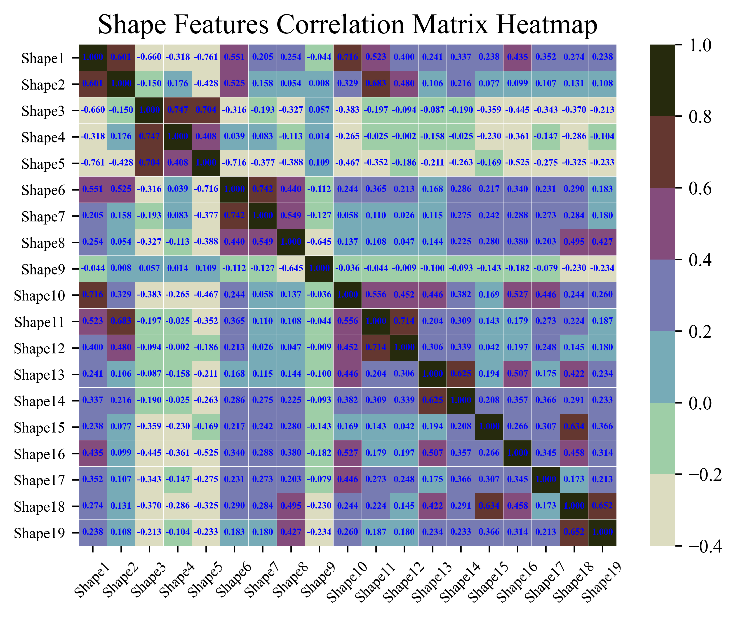


**Figure S2** The correlation of selected (**a**) clinical data, (**b**) texture features and (**c**) shape features after statistical analysis in heatmap. Owing to the limitation of figures, the texture features were described briefly as the combination of Texture and the order of them shown in Additional file 1 Table S6. Shape features were described in the same way.
